# Risk Factors for Cervical Hematoma After Thyroid Surgery at a Portuguese Hospital: A Retrospective Study

**DOI:** 10.7759/cureus.72928

**Published:** 2024-11-03

**Authors:** Inês Dinis, Cândida Sofia Pacheco Pereira, Catarina Ferros, Carla Pereira

**Affiliations:** 1 Anesthesiology, Unidade Local de Saúde (ULS) de Viseu Dão-Lafões, Viseu, PRT

**Keywords:** anesthesiology practice, cervical hematoma, outpatient anesthesia, post thyroidectomy, thyroidectomy complications

## Abstract

Objective: Cervical hematoma post thyroid surgery is an uncommon but potentially life-threatening postoperative complication. Moreover, despite its low incidence, this complication has been a barrier to outpatient surgical care. Assessing postoperative complication rates and their risk factors can improve the safety and cost-effectiveness of these procedures, which is especially useful in promoting outpatient surgical care.

Methodology: This retrospective study included 198 patients who underwent thyroid surgery in the district of Viseu (Portugal) between January 2020 and December 2021. The sample was analyzed to determine the incidence of cervical hematoma post thyroid surgery and to identify possible medical and modifiable anesthetic risk factors related to hematoma. To determine the risk factors, the sample was divided into two groups: Group 1 included patients without postoperative cervical hematoma and Group 2 included patients with postoperative cervical hematoma

Result: The incidence of postoperative cervical hematoma was 6.0%, slightly higher than previously reported in the literature. Univariate analysis identified two factors associated with post-surgical cervical hematoma in our sample: a medical history of obstructive lung disease and intraoperative administration of ketorolac. Several studies have reliably associated a history of chronic obstructive pulmonary disease (COPD) with airway reactivity, especially during an anesthetic emergency. The use of non-steroidal analgesics is widespread in intraoperative practice, and the literature consistently supports the safety of their administration in endocrine cervical surgical procedures. Our study revealed controversies related to this topic, and in our opinion, patients who received intraoperative ketorolac in our cohort tended to follow an opioid-free analgesia regimen, leading to more pain and discomfort post surgery. Discomfort and pain in the immediate postoperative period have been identified as potential risk factors for cervical hematoma.

Conclusion: Patients with obstructive lung disease ​​​​​​should be carefully monitored after thyroid surgery and may not be suitable for an ambulatory thyroidectomy. The risk of postoperative bleeding and the benefits of intraoperative ketorolac administration should be balanced, particularly for high-risk patients like those with obstructive pulmonary disease. The establishment of standardized anesthetic protocols for thyroid surgery can enhance the safety and cost-effectiveness of procedures, helping to define feasible preoperative criteria for patient selection in outpatient care.

## Introduction

Thyroid surgery refers to thyroid tissue resection and may include thyroidectomy or hemithyroidectomy. Despite the thyroid gland being a highly vascularized organ, the procedure is safe and commonly performed for the treatment of both benign and malignant tumors, autoimmune disorders, and compressing airway problems. [1]

The number of such surgeries performed on an outpatient basis is growing in developed countries, but the occurrence of complications is a barrier to current practice worldwide, and many are due to modifiable risk factors, such as human factors [2,3]. Cervical hematoma post thyroid surgery is an uncommon (incidence of 0-4%), unpredictable, and potentially life-threatening early complication (first 24 hours), as it may result in airway obstruction [1]. The fear of this adverse event leads most surgical centers to conservative postoperative management and inpatient surgical procedures. This is a common indication for reoperation, whether it's an emergency or not. Depending on the airway compromise and the speed of its onset, it can turn into a front-of-neck airway (FONA) scenario [4]. Concerning the pathophysiology it is believed that compression results in venous and lymphatic drainage systems blockage and consequent laryngopharyngeal edema and airway obstruction [2,4,5].

Over the past few years, there have been reports of post-surgical thyroid bleeding risk factors, which include thyroid diseases such as Graves' disease and toxic nodular goiter, as well as patient and surgical characteristics [5,6]. Thyroid reoperation, associated with scaring and fibrosis, is also a known associated risk [5-10]. Also, other conditions have been associated with postsurgical hematoma formation, such as the use of drainage, and medical priors: the use of antiplatelets or anticoagulants, bleeding disorders, and hypertension [8,9,11-14]. However, there is a lack of studies reporting the risk factors associated with anesthetic techniques.

The aim of this study is to determine the incidence of postoperative cervical hematoma following thyroid surgery and to identify the medical and anesthetic risk factors associated with it.* *The answers to these questions can help surgical centers expand thyroid surgery to include outpatient procedures.

## Materials and methods

Study design

This was a retrospective non-randomized cohort study conducted at São Teotónio Hospital located in the city of Viseu, Portugal. The following inclusion criteria were applied: adult patient (aged 18 or over), preoperative euthyroid state, who underwent elective thyroid surgery (total thyroidectomy or thyroid lobectomy), performed by a group of surgeons with several years of experience in thyroid surgery, between January 1, 2020, and December 31, 2021. The sample of 198 patients was divided into two groups: 12 patients had hematoma following thyroid surgery and 186 patients did not. 

Data sources

Data were obtained from the hospital database (SClinico), anesthetic records, and laboratory and diagnostic testing reports. Patient demographic and clinical variables included were age, gender, and comorbidities: arterial hypertension, dyslipidemia (DLP), atrial fibrillation (AF), antithrombotic agent use, smoking status, chronic obstructive lung disease (COPD), diabetes mellitus (DM), and stroke history. Laboratory variables included coagulation time (activated partial thromboplastin time (APTT), prothrombin time (PT)) and hemogram values (platelets, hemoglobin). Anesthetic variables included the type of general anesthesia (volatile agents for maintenance of anesthesia or total intravenous anesthesia), use of nerve integrity monitoring (NIM),​​​​​ and the agents of ​​intraoperative adjuvant analgesia (tramadol, ketorolac, acetaminophen, ketamine, parecoxib​​​​​​, metamizole, and morphine​). Procedure variables included surgery type (thyroidectomy total and thyroid lobectomy), surgery duration, indications for surgery (malignant), drain placement, the weight of the thyroid tissue after resection, other postoperative complications (dysphonia, hypocalcemia), and the occurrence of cervical hematoma and its surgical drainage time.

Data were analyzed using the IBM SPSS Statistics for Windows, Version 24.0, (Released 2016; IBM Corp., Armonk, New York, United States), and patient demographic characteristics and clinical information were summarized. The chi-square test (alternative Fisher’s exact test) and Mann-Whitney U test were used for testing statistical differences between groups. Logistic binary regression was applied to predict the occurrence of cervical hematoma after thyroid surgery and logistic regression coefficients were used to estimate the odds ratio for each of the independent variables in the model. The absence of perfect multicollinearity among the independent variables was ensured. A p-value less than 0.05 was considered statistically significant.

## Results

In total, 198 elective thyroid surgeries were performed between January 2020 and December 2021. Thyroid lobectomy was the most performed surgery, in 101 (51%) cases. In all cases, general anesthesia was used: 35 (68.2%) with volatile agents for maintenance of anesthesia and 63 (31.8%) totally intravenous anesthesia (TIVA). In addition to the American Society of Anesthesiologists (ASA) monitoring, nerve Integrity monitor (NIM) was used in 28 (14.4%) patients. Intraoperatively, the most used analgesic drugs in descending order were paracetamol, tramadol, parecoxib, remifentanil, metamizole and ketorolac. Regarding the histological assessment, the mean weight of the excised thyroid tissue was 36 grams (SD 32) and the anatomopathological diagnoses were mostly benign, in 112 (56.6%) cases. 

Baseline characteristics of patients, anesthesia, and surgery are shown in Table 1. In the postoperative period, cervical hematoma had an incidence of 6% (n=12). All cervical hematomas were surgically drained: 11 (91.7%) were re-operated within the first 24 hours postoperatively, and one (8.3%) was re-operated after 72 hours. Univariate analysis identified statistical differences between Group 1 and Group 2 (Table 1). Patients in Group 2, who had cervical hematomas, had a higher proportion of a medical history of COPD and were more likely to receive ketorolac intraoperatively compared to those in Group 1.

**Table 1:** Differences between the groups with and out cervical hematoma postoperatively after thyroid surgery ᶲ - Fisher´s exact test, ¥ - Qui-square test

Surgery		Cervical Haematoma						
		No (n=186)		Yes (n=12)		Total (n=198)		
		Count	Percentage	Count	Percentage	Count	Percentage	p-value
Thyroid Surgery	Thyroid lobectomy	93	50.0	8	66.7	101	51.0	0.263^¥^
	Total thyroidectomy	93	50.0	4	33.3	97	49.0	
Gender	Male	47	25.3	6	50.0	53	26.8	0.088^ᶲ^
	Female	139	74.7	6	50.0	145	73.2	
Arterial Hypertension	No	103	55.4	6	50.0	109	55.1	0.717^¥^
	Yes	83	44.6	6	50.0	89	44.9	
Dyslipidemia	No	122	65.6	7	58.3	129	65.2	0.756^ᶲ^
	Yes	64	34.4	5	41.7	69	34.8	
Diabetes mellitus	No	165	88.7	11	91.7	176	88.9	1^¥^
	Yes	21	11.3	1	8.3	22	11.1	
Chronic obstructive pulmonary disease	No	176	94.6	9	75.0	185	93.4	0.035^ᶲ^
	Yes	10	5.4	3	25.0	13	6.6	
Obstructive sleep apnea	No	178	95.7	12	100.0	190	96.0	1^¥^
	Yes	8	4.3	0	0.0	8	4.0	
Smoke status	No	177	95.7	10	90.9	187	95.4	1^¥^
	Yes	8	4.3	1	9.1	9	4.6	
Antiplaquets Drug used	No	175	94.1	11	91.7	186	94.9	0.476^¥^
	Yes	9	5.9	1	8.3	10	5.1	
Antithrombotic Drug used	No	182	94.3	11	91.7	193	97.4	0.271^¥^
	Yes	4	5.7	1	8.3	5	2.6	
Malignant tumor	No	105	97.8	7	58.3	112	56.6	1^ᶲ^
	Yes	81	2.2	5	41.7	86	43.4	
Drain used	No	52	29.7	4	33.3	56	29.9	1^ᶲ^
	Yes	123	70.3	8	66.7	131	70.1	
Nerve integrity monitor (NIM) used	No	162	95.3	8	66.7	170	85.8	0.071^¥^
	Yes	24	4.7	4	36.4	28	14.4	
Balanced anesthesia	No	56	30.1	7	58.3	63	31.8	0.056^¥^
	Yes	130	69.9	5	41.7	135	68.2	
Remifentanil used	No	102	60.4	5	41.7	107	59.1	0.234^ᶲ^
	Yes	67	39.6	7	58.3	74	40.9	
Ketorolac	No	152	89.9	8	66.7	160	88.4	0.036^ᶲ^
	Yes	17	10.1	4	33.3	21	11.6	

Binary logistic regression revealed that patients with a history of COPD had 7.17 times higher odds of developing a cervical hematoma after thyroid surgery compared to those without COPD. Additionally, patients who received ketorolac intraoperatively had 5.26 times higher odds of developing a cervical hematoma compared to those who did not receive ketorolac. However, only 13% of the variation in the dependent variable can be explained by the independent variables (pseudo-R² of Nagelkerke = 0.132). The details are given in Table 2.

**Table 2:** Predictors of cervical hematoma

Predictors of cervical hematoma	B	S.E.	Wald	df	Sig.	Exp(B)=OR	95% CI for EXP(B)	
							Lower	Upper
Chronic obstructive pulmonary disease (1)	1.970	.787	6.267	1	.012	7.170	1.534	33.523
Ketorolac (1)	1.660	.696	5.693	1	.017	5.258	1.345	20.557
Constant	-3.244	.423	58.751	1		.039		

## Discussion

The incidence of thyroid cancer is increasing in developed countries, resulting in a negative socioeconomic impact on national healthcare systems. Traditionally, thyroid surgery has been considered a relatively low-risk inpatient procedure. However, some countries have developed outpatient hemithyroidectomy protocols, which have been reported as safe and feasible, without a rise in postoperative complications such as cervical hematoma, dysphonia, or hypocalcemia. These complications, while uncommon, can be life-threatening and may occasionally require re-intervention or re-admission [15-17].

Understanding the trends in postoperative complication rates can assist ambulatory surgery centers in developing effective patient selection criteria for outpatient surgical care [15-17].

Postoperative cervical hematoma is a particularly concerning complication following thyroid surgery, as it is associated with significant morbidity and mortality. Most cases of postoperative cervical hematoma occur within the first 24 hours, with the majority arising in the first six hours. Therefore, anesthesiologists are often the first to manage these complications, primarily in the post-anesthetic care unit (PACU) [2,8]. Prompt assessment and management are required, occasionally necessitating bedside hematoma evacuation with or without emergency tracheal intubation [1,4].

At our hospital, thyroid surgical procedures are performed exclusively in an inpatient setting. The current study found the incidence of cervical hematoma to be 6%, which is slightly higher than the 1-4% range reported in the existing literature [1,6-8]. Also, only two medical factors were associated with cervical hematoma: a history of COPD and intraoperative administration of ketorolac.

Pre-existing patient and procedural characteristics have been identified as risk factors for cervical hematoma following thyroid surgery. These include male gender, history of alcohol consumption, smoking history, larger thyroid volume, extent of dissection, type of hemostatic techniques used, use of drainage devices, and the expertise of the surgical team [4-8,10,12-14].

COPD is characterized by persistent airflow limitation that is usually progressive and associated with an enhanced chronic inflammatory response in the airways and the lungs to noxious particles or gases [18]. The association may be due to increased airway reactivity and more coughing during an anesthetic emergency. In fact, increased airway pressures can contribute to higher venous pressure or venous congestion resulting in a higher risk of bleeding, due to vascular rupture [5,19].

Ketorolac is a nonsteroidal anti-inflammatory drug (NSAID) that is used as part of a multimodal postoperative analgesic regimen. It offers significant analgesic benefits, particularly in an outpatient setting, as it can provide opioid-free pain relief and support faster patient recovery and discharge [20]. Previous meta-analyses on the use of ketorolac have failed to demonstrate a clinically significant increase in the risk of postoperative bleeding [20]. The risk of bleeding associated with a single intraoperative bolus of ketorolac varies across different types of surgeries, including those with high bleeding risk, and is not well-defined in the literature [21-24]. Despite this, our study found that patients who received ketorolac during surgery were significantly more likely to develop cervical hematoma after thyroid surgery. This result should be interpreted with caution due to the small sample size of cervical hematoma cases (n=12), which affects both internal and external validity. Recent prospective studies have reported that a higher numeric rating scale (NRS) score for postoperative pain and the administration of two or more doses of ketorolac postoperatively may be independent risk factors for post-thyroidectomy cervical hematoma [25]. Additionally, only 5% of patients who received ketorolac intraoperatively also received opioid analgesia (tramadol), compared to 21% of patients who did not receive ketorolac.

Current medical literature associates thyroid surgical procedures with mild to moderate postoperative pain; however, there is limited strong scientific evidence regarding the optimal postoperative analgesic regimen [26,27]. The primary sources of pain related to thyroidectomy are the incision site and the throat. However, patients have also reported pain in other areas, such as the neck, beyond the incision site [26,27].

These issues are of utmost importance if we are to transition thyroid surgeries to outpatient settings, where pain and complications must be monitored more closely.

Several limitations should be acknowledged in the present study. First, this was a retrospective cohort study without randomization. Secondly, factors such as the surgical technique (e.g., methods of hemostasis like cold dissection or electrocautery), the surgeon's expertise, and intraoperative blood pressure were not recorded in the database, which may act as confounding variables. Thirdly, the data relied on records from various anesthetists, leading to variable accuracy.

## Conclusions

Cervical hematoma after surgery is an uncommon but potentially life-threatening postoperative complication. To prevent postoperative cervical hematoma following thyroid surgery, it is essential to identify high-risk patients, such as those with a history of COPD, who require close monitoring during the perioperative period. Another modifiable risk factor for cervical hematoma is the analgesic regimen. The lack of standardized guidelines for managing acute pain in perioperative settings has led to a heterogeneous approach and conflicting reports.

Our study showed that patients who received ketorolac were more prone to cervical hematoma after thyroid surgery. This may be due to these patients experiencing more discomfort and pain following surgery, as they typically use an opioid-free analgesic regimen. There are inconsistencies regarding pain scores after thyroid surgery. Standardizing analgesic regimens using a multimodal approach could enhance the safety and cost-effectiveness of these common surgeries. Additionally, identifying trends in postoperative complications can help ambulatory surgery centers develop effective and safe protocols and establish patient selection criteria.
